# PPARγ agonist through the terminal differentiation phase is essential for adipogenic differentiation of fetal ovine preadipocytes

**DOI:** 10.1186/s11658-017-0037-1

**Published:** 2017-03-23

**Authors:** Yong Pu, Almudena Veiga-Lopez

**Affiliations:** Department of Animal Science, Michigan State University, 474 S. Shaw Lane Rm 1230 F, East Lansing, MI 48824 USA

**Keywords:** Sheep, Fetal, Preadipocyte, Adipogenic differentiation

## Abstract

**Background:**

Although the 3T3-L1 preadipocyte cell line represents an informative model for *in vitro* adipogenesis research, primary cultured cells are often needed to understand particular human or animal metabolic phenotypes. As demonstrated by *in vitro* cultured preadipocytes from large mammalian species, primary cultured cells require specific adipogenic differentiation conditions different to that of the 3T3-L1 cell line. These conditions are also species-specific and require optimization steps. However, efficient protocols to differentiate primary preadipocytes using alternative species to rodents are scarce. Sheep represent an amenable animal model for fetal biology and developmental origins of health and disease studies. In this work, we present with the first detailed procedure to efficiently differentiate primary fetal and adult ovine preadipocytes.

**Methods:**

Fetal and adult ovine adipose and skin tissue harvest, preadipocyte and fibroblast isolation, proliferation, and standardization and optimization of a new adipogenic differentiation protocol. Use of commercial cell lines (3T3-L1 and NIH-3T3) for validation purposes. Oil red O stain and gene expression were used to validate adipogenic differentiation. ANOVA and Fisher’s exact test were used to determine statistical significance.

**Results:**

Our optimized adipogenic differentiation method included a prolonged adipogenic cocktail exposure time from 2 to 8 days, higher insulin concentration, and supplementation with the peroxisome proliferator-activated receptor gamma (PPARγ) agonist, rosiglitazone. This protocol was optimized for both, fetal and adult preadipocytes.

**Conclusions:**

Our protocol enables successful adipogenic differentiation of fetal and adult ovine preadipocytes. This work demonstrates that compared to the 3T3-L1 cell line, fetal ovine preadipocytes require a longer exposure to the differentiation cocktail, and the need for IMBX, dexamethasone, and/or the PPARγ agonist rosiglitazone through the terminal differentiation phase. They also require higher insulin concentration during differentiation to enhance lipid accumulation and similar to human primary preadipocytes, PPARγ agonist supplementation is also required for ovine adipogenic differentiation. This work highlights species-specific differences requirements for adipogenic differentiation and the need to develop standardized methods to investigate comparative adipocyte biology.

## Introduction

To study adipogenesis, adipogenic differentiation, and their regulation, investigators often resource to the commercially available preadipocyte cell line 3T3-L1 of murine origin [1]. Results from these studies are not as useful for applications on human health as expected, given the physiological and metabolic differences among species [2]. Human primary cultured cells are the best resource to understand human adipocyte biology in deranged metabolic states [2], but cannot inform about cell events that occur during prenatal life. The developmental origins of health and disease theory is pointing to early life events as one of the contributing factors towards the increased prevalence of metabolic diseases worldwide, including obesity [3–5]. To test hypotheses relative to fetal adipose tissue differentiation and fate and/or the effects of prenatal exposures (chemicals, stress, nutrition) on adult metabolic risk, alternative models are often needed [6]. Other large mammalian species, such as feline and porcine primary cells, have recently been used as alternatives [7, 8]. The sheep is an outstanding animal model to understand how prenatal exposures can affect adult metabolic disease risk [9–12] because, as humans, sheep are a monotocous and precocial species, with majority of organs maturing before birth [13], including the adipose tissue [14]. Identified barriers to the advance of our understanding on adipocyte biology include the great diversity of protocols available for some cell lines and the lack of standardized methods of adipogenic differentiation in other mammalian species [2]. As we demonstrate in this study, standard murine 3T3-L1 differentiation protocols [15, 16] are not suitable for ovine preadipocyte adipogenic differentiation.

Adipocyte differentiation is a complex process by which preadipocytes transition into lipid-filled, insulin-responsive adipocytes. Adipocyte fate is controlled by transcription factors, including peroxisome proliferator-activated receptor gamma (PPARγ), CCAAT/enhancer-binding proteins (C/EBPs), and sterol regulatory element binding protein (SREBP). Preadipocyte differentiation is routinely initiated by a 48 h exposure to a basic 3T3-L1 differentiation induction cocktail containing 3-isobutyl-1-methyxantine (IBMX), dexamethasone, and insulin [17]. IBMX regulates C/EBPβ [18], enhances 3T3-L1 differentiation upon longer exposures [15], and alone [19] or in combination with dexamethasone [20], regulates PPARγ activity. PPARγ, the master regulator of adipogenesis, cooperates with C/EBPα to initiate adipogenic differentiation, is required in human preadipocyte differentiation [21, 22], and can increase glycerol-3-phosphate dehydrogenase (GPDH) activity in sheep preadipocytes [23], an enzyme involved in lipid biosynthesis. PPARγ agonists, such as thiazolidinediones, have been used to enhance adipogenic differentiation in 3T3-L1 and primary preadipocytes of large mammals [7, 14, 17]. In addition, supplementation with insulin promotes preadipocyte differentiation [24], but the insulin dose is among the most variable media components of 3T3-L1 differentiation protocols [17].

In this work, we aimed to standardize a protocol for effective ovine adipogenic differentiation using fetal and adult primary cultured preadipocyte that will help understand basic adipocyte biology knowledge in a relevant meat-producing species, as well as events occurring during fetal adipose tissue remodeling. To enable ovine adipogenic differentiation, we applied several media modifications. Here, we have only presented the key modifications that resulted in significant improvements in ovine preadipocyte adipogenic differentiation.

## Materials and methods

### Fetal and adult tissue harvest

All procedures used in this study were approved by the Institutional Animal Care and Use Committee of Michigan State University (MSU) and are consistent with the National Research Council’s Guide for the Care and Use of Laboratory Animals and the Animal Welfare Act. The study was conducted at the MSU Research Facility (East Lansing, MI; 42.7° N, 84.4° W) using an in-house flock of multiparous Polypay x Dorsett breed of sheep. Female sheep were bred using a time mated pregnancy strategy. At gestational day 120 and after humane sacrifice, fetal perirenal adipose tissue and skin from the abdominal midline were harvested from female fetuses to isolate fetal ovine preadipocytes (oPADs) and skin fibroblasts. Immediately after, tissues were placed in transport medium (Omental Preadipocyte Medium, Zen Bio, USA) and processed within two hours. Adipose tissue was also collected from the subcutaneous adipose depot of three adult females after humane sacrifice.

### Generation of fetal and adult ovine primary preadipocytes

Preadipocyte isolation method was conducted following a common standard protocol used in isolation of human adipogenic precursor cells [25]. This isolation protocol followed by adipogenic differentiation allows an average cell differentiation rate of 50-60% [25]. In brief, fetal and adult oPADs were isolated by rinsing a 1–2 g of adipose tissue with pre-warmed Dulbecco’s phosphate-buffered saline (DPBS) with antibiotic-antimycotic (Invitrogen), removing any visible blood clots and connective tissue using sterile forceps and then minced into small pieces. Collagenase-I (C0130, Sigma, 1 mg/ml) was used to digest tissue for 40 min in a 37 °C water bath. The mixture was filtered through a mesh filter (250 μm, U-CMYBK-250, Component Supply Company, Fort Meade, USA) and centrifuged at 1,200 rpm for 5 min. The cell pellet was washed with fresh omental preadipocyte medium and seeded into 6-well plates. After six days of culture, cells were frozen (Preadipocyte Cryopreservation Medium, FM-1-100, Zen-Bio) and stored in liquid nitrogen until further use. All experiments were performed using fetal oPAD primary cells (passage 3) from one female and validated with fetal and adult oPADs from 2 and 3 additional females, respectively. The 3T3-L1 (preadipocyte) and NIH-3T3 (fibroblastic) cell lines were used as positive and negative control of adipogenic cells, respectively. Ovine fetal primary fibroblasts were also used as a negative control of adipogenesis.

### Generation of fetal ovine primary skin fibroblasts

Ovine fetal primary fibroblasts were isolated as follows. After hair and connective tissue removal using sterile forceps, fetal skin tissue was rinsed twice with pre-warmed DPBS supplemented with antibiotic and antimycotic. Tissue (1 g) was minced into small pieces and distributed into 6-well plates with 0.2 ml of serum per well. After 6 h incubation, 2 ml of basal medium consisting of DMEM/F12 medium (Invitrogen) supplemented with 1% penicillin-streptomycin, 10 mM HEPES, and 10% fetal bovine serum (Corning, Manassas, VA, USA) was added into the plate and primary cells cultured for 4 to 6 days until confluency. Cells were then trypsinized and passaged as primary fibroblasts for further study.

### Ovine primary preadipocytes proliferation

Primary cultured oPADs were trypsin digested (Invitrogen, 0.05%) for subculture upon 90% confluency. Proliferation ability for each cultured cell (passage 3) was assessed by growth curve analysis. Cells were seeded at a density of 10,000 cells per well in 24-well plates and the counts were performed in haemocytometer chamber. Cell counts were performed every 24 h for 8 days. Triplicate wells for each time point per cultured cell were used.

### Cell culture and adipocyte differentiation

**Fig. 1:**
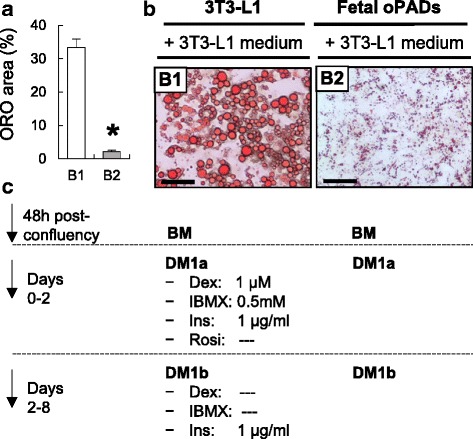
Differentiated 3T3-L1 cells and fetal ovine preadipocytes (oPADs) with 3T3-L1 medium. Quantification of ORO positive area (mean ± SEM; **a**), representative ORO stain images of 3T3-L1 (*B1*) and fetal oPADs (*B2*) after 8 days of differentiation (**b**), and differentiation medium details (**c**). Scale bar: 50 μm. *BM:* basal medium, *Dex*: dexamethasone, *DM*: differentiation medium, *Ins*: insulin, *oPADs:* ovine preadipocytes, *Rosi*: rosiglitazone. Three fetal oPAD primary cells (passage 3) from three different fetuses were used. Asterisk represents significant differences (*P* < 0.05)

**Fig. 2:**
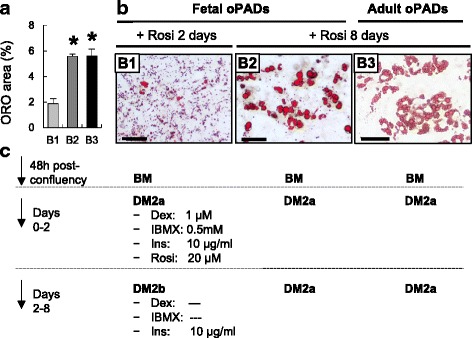
Effect of rosiglitazone supplementation and prolonged adipogenic cocktail exposure on ovine fetal and adult preadipocyte (oPADs) differentiation. Quantification of ORO positive area (mean ± SEM; **a**), representative ORO stain images of fetal (*B1* (only insulin supplementation in days 3 to 8); *B2* (optimized medium)) fetal and adult oPADs (*B3*; optimized medium) after 8 days of differentiation (**b**), and differentiation medium details (**c**). Scale bar: 50 μm. *BM:* basal medium, *Dex*: dexamethasone, *DM*: differentiation medium, *Ins*: insulin, *oPADs:* ovine preadipocytes, *Rosi*: rosiglitazone. Three fetal oPADs and three adult oPADs primary cells (passage 3) from three different fetuses and three different adult sheep, respectively, were used. Asterisks represent significant differences (*P* < 0.05)

**Fig. 3:**
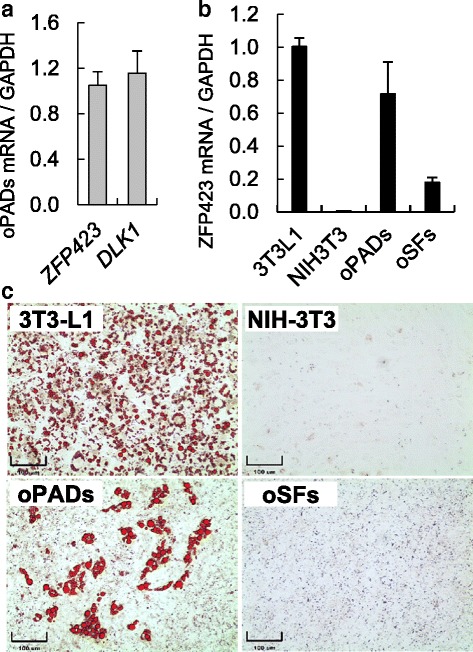
Preadipocyte and adipogenic markers mRNA expression in murine and ovine primary cultured cells and cell lines. **a**) DLK1 and ZFP423 mRNA expression in ovine fetal preadipocytes (oPADs). **b** ZFP423 mRNA expression in undifferentiated 3T3-L1 and NIH-3T3 cell lines and fetal ovine primary cultured cells, oPADs and fetal skin fibroblasts (oSFs). **c** Oil red O stain of 3T3-L1 cell line (preadipocyte), NIH-3T3 cell line (fibroblast), ovine female fetal preadipocytes (oPADs), and skin fibroblasts (oSFs) after differentiation induction for 8 days. Three fetal oPADs and three fetal oSFs primary cells (passage 3) from three different fetuses were used

Before differentiation induction, fetal oPADs (passage 3), 3T3-L1 cells (American Type Culture Collection, ATCC, Manassas, VA, USA; within passage 6), and NIH-3T3 (ATCC, Manassas, VA, USA; within passage 6) were cultured in basal medium (same as for oSFs) until confluency and allowed to grow for two additional days. Thereafter, adipocyte differentiation was induced. Summary of adipogenic differentiation media and exposure times are detailed in Figs. 1c and 2c. 3T3-L1 differentiation medium 1a (DM1a) consisted of basal medium supplemented with biotin (33 μM), pantothenate (17 mM), insulin (1 μg/ml), dexamethasone (1 μM) and 3-isobutyl-1-methylxanthine (IBMX, 0.5 mM). 3T3-L1 differentiation medium 1b (DM1b) was same as DM1a, without dexamethasone and IBMX. 3T3-L1 were exposed to DM1a from days 0 to 2 and to DM1b from days 3 to 8 (Fig. 1c). All media were replaced every 48 h. Composition of the modified differentiation medium 2a (DM2a) was the same as DM1a, but supplemented with increased insulin concentration (10 μg) and rosiglitazone (20 μM). Differentiation medium 2b (DM2b) was the same as DM2a, but without dexamethasone, IBMX, and rosiglitazone (Fig. 2c). Rosiglitazone concentration was based on previous reports [26] and pilot work in our laboratory (data not shown). Ovine female fetal skin fibroblasts were induced to differentiate using the final differentiation protocol to demonstrate how ovine fetal fibroblasts lack the ability to differentiate upon exposure to the PPARγ agonist, rosiglitazone (Fig. 3c). 3T3-L1 and NIH-3T3 cell lines were also induced to differentiate (Fig. 3c) in DM1a media for 2 days, following by DM1b media for additional 6 days.

### Oil Red O stain

Preadipocyte differentiation into mature adipocytes was determined by Oil Red O (ORO) stain, as a marker of lipid accumulation. In brief, ORO (0.3%) was dissolved in isopropanol diluted in water (0.18%). Adipocytes at differentiation day 8, were washed with DPBS twice then fixed in 10% formalin for 30 min at room temperature. Cells were washed with DPBS and incubated 5 min in 60% isopropanol followed by incubation in ORO working solution. After multiple DPBS washes, bright field images were captured on an inverted microscope. Five images of each treatment were used to calculate ORO positive area using the Image J software [27].

### Quantitative real time PCR (qRT-PCR)

To evaluate the dynamic expression of genes involved in adipogenic differentiation, qRT-PCR (ABI-Quant Studio 7 Flex Real-Time PCR System, Thermo Fisher, Carlsbad, CA) was performed to examine the mRNA expression of ovine genes encoding for peroxisome proliferator-activated receptor gamma (*PPARɣ*), fatty acid binding protein 4 (*FABP4*), CCAAT/enhancer-binding protein alpha (*C/EBPα*), adiponectin (*ADIPOQ*), protein delta homolog 1 (*DLK1*) and zinc finger protein 423 (*ZFP423*). Primer sequences are shown in Table 1. The sizes of the DNA templates were confirmed by 1.5% agarose gel. Cultured cells were washed three times with ice cooled DPBS and total RNA extracted with an RNeasy Mini kit (Qiagen, Hilden, Germany) according to the manufacturer’s protocol. RNA quality and concentration were measured by Nanodrop (Thermo Fisher Science, Wilmington, NC, USA). 1 μg RNA (quality: A260/A280: 2.0 ± 0.05; concentration: 200 ± 50 ng/μl) was reverse transcribed into cDNA using a High Capacity cDNA Reverse Transcription Kit (Promega, Madison, WI, USA) in 10 μl reaction volume. cDNA amplification reaction (50 ng) consisted of template denaturation and polymerase activation at 95 °C for 30 s, followed by 40 cycles of denaturation at 95 °C for 15 s, annealing at 60 °C for 30 s, and extension at 72 °C for 30 s. All experiments and qRT-PCR were run in triplicate. Melt curve analyses were performed for all genes, and the specificity as well as integrity of the PCR products was confirmed by the presence of a single peak. The levels of mRNA encoding the indicated genes were normalized against three housekeeping genes *GAPDH, RPL27, and β-actin* to calculate relative fold change to that of the control. Results using all three housekeeping genes provided with the same results, but only *GAPDH* data are shown.

**Table 1:** Primers for quantitative real time PCR

Gene	Primer	Length (bp)	Accession number
*oADIPOQ* - Forward	GGAGATCCAGGTCTCGTTGG	98	NM_001308565
*oADIPOQ* - Reverse	TTTCTGCCTGGGACTCCTGG		
*oβ-ACTIN -* Forward	CCAACCGTGAGAAGATGACC	97	NM_001009784
*oβ-ACTIN -* Reverse	CCAGAGGCGTACAGGGACAG		
*oC/EBPα* - Forward	CCCCGACAGGAGCAAGGT	114	KF830871
*oC/EBPα* - Reverse	GGTTCAAAGCCCCAAGT		
*oDLK1* - Forward	GGCATCGTCTTCCTCAAC	89	XM_015102053
*oDLK1* - Reverse	CGCAGCAGCAGATTCTTC		
*oFABP4* - Forward	GGATGATAAGCTGGTGCTGG	53	NM_001114667.1
*oFABP4* - Reverse	CTCTGGTAGCAGTGACACCG		
*oGAPDH* - Forward	TTCCACGGCACAGTCAA	241	NM_001190390
*oGAPDH* - Reverse	TCACGCCCATCACAAAC		
*mGAPDH* - Forward	CGGCAAATTCAACGGCACA	84	NM_001289726
*mGAPDH* - Reverse	TCTCGCTCCTGGAAGATGG		
*oPPARγ* - Forward	TGGATGACCACTCCCATGCC	97	NM_001100921
*oPPARγ* - Reverse	TTGGGAACGGAATGTCCTC		
*oRPL27 -* Forward	CGCAAGGCCCGACGAGAGGC	93	XM_015098799
*oRPL27 -* Reversr	GACCTAAAACCGCAGCTTCTGG		
*oZFP423* - Forward	CCCGATTCCAGCAACCACA	160	XM_015100428
*oZFP423* - Reverse	CGTCATCCCGCATCTTCTTCT		
*mZFP423* - Forward	CCGTCTGCTTCACAGTCTTCG	155	NM_001310520
*mZFP423* - Reverse	TGCGTGCTGGCTCATCG		

Note: o refers to ovine, m refers to murine. Accession number from NCBI gene database

### Statistics

All data are presented as mean ± SEM. All experiments (adipogenic differentiation in fetal and adult oPADs and oSFs, and qRT-PCR) were conducted in triplicate. Appropriate transformations were applied, as needed, to account for normality of data. ANOVA was used to compare gene expression data among differentiation time points followed by Dunnett’s posthoc test. For comparing percent differences Fisher’s exact test was used. Statistical software used was PASW Statistics for Windows release 18.0.1. Differences were considered significant at *P* < 0.05.

## Results and discussion

**Fig. 4:**
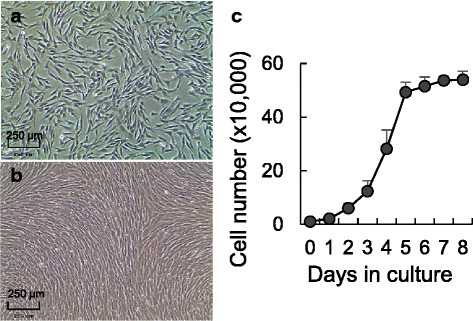
Characterization of primary fetal ovine preadipocytes (oPADs). oPADs in culture (**a**) and at confluency (**b**). **c** Preadipocytes growth curve (mean ± SEM) over 8 days (three fetal oPADs cell lines)

In this work, we have successfully established a protocol to induce adipogenic differentiation in fetal and adult ovine primary cultured preadipocytes. As expected from preadipocytes, the morphology of oPADs was phenotypically fibroblastic (Fig. 4a-b), demonstrated good proliferative ability (Fig. 4c), and expressed both, the preadipocyte marker (*DLK1*) [28] and the adipogenic differentiation ability marker (*ZFP423*) [29] mRNA (Fig. 3a). *ZFP423* mRNA expression was highest in the 3T3-L1 cell line (100%, preadipocyte cell line), followed by oPADs (71%), ovine fetal primary fibroblasts (17%) and NIH-3T3 cell line (0.5%, fibroblast cell line) and was positively correlated with the differentiation ability of each of these cultured cells and cell lines (Fig. 3b-c).

We first tested the ability of the 3T3-L1 differentiation protocol (Fig. 1c) to induce differentiation in fetal oPADs and compared it to the differentiation ability in the 3T3-L1 cell line (Fig. 1). After 8 days of differentiation, over 90% of 3T3-L1 cells differentiated in fully mature adipocytes as shown by ORO stain (Fig. 1a and b1). However, the 3T3-L1 protocol was not able to induce terminal adipogenic differentiation in oPADs (Fig. 1a and b2). The ≥ 16-fold difference between 3T3-L1 and fetal oPADs differentiation was similar to reports standardizing adipogenic differentiation with human primary preadipocytes [22, 30].

Because insulin concentration is among the adipogenic media supplements with the largest variation [17], we tested if increasing the insulin concentration would improve accumulation of lipid droplets during adipogenic differentiation. A 10-fold increase in insulin concentration (10 μg/ml) induced the formation of small and middle-sized lipid droplets in fetal oPADs (data not shown). 3T3-L1 cells do not require adipogenic induction with a PPARγ agonist, however differentiation protocols for large mammalian species [7, 8, 14] preadipocyte and human mesenchymal stem cells into adipocytes often include this modification [31, 32]. To test if supplementation with a PPARγ agonist results in enhanced fetal ovine preadipocyte differentiation, rosiglitazone was selected over other PPARγ agonists, such as troglitazone, because troglitazone is only a partial PPARγ agonist [33]. Rosiglitazone (20 μM) was included into the differentiation medium (DM2a; Fig. 2c) and cells exposed for 2 days. Thereafter, differentiation medium was replaced with DM2b (Fig. 2c) and fetal oPADs cultured for 6 additional days. Addition of a PPARγ agonist for 48 h improved differentiation, increasing medium-sized lipid droplet formation (Fig. 2b1) and suggesting that similar to feline [7], porcine [34], and human [31] preadipocytes, ovine preadipocytes have lower sensitivity to insulin than murine cell lines and PPARγ agonist may be a more stringent requirement for adipogenic differentiation in these species.

Since previous studies have shown that longer exposure (>2 days) to the differentiation cocktail can enhance adipogenesis [35, 36], we tested if a longer exposure to the DM2a increased ovine adipogenic differentiation. Exposure to the DM2a for 8 days resulted in an enhanced adipogenic differentiation of oPADs and formation of medium to large sized lipid droplets (Fig. 2b2). With this differentiation protocol, lipid accumulation is readily visible under light microscope starting at day 3 of culture. The final protocol (8 days of DM2a exposure) was tested in two additional fetal oPADs confirming differentiation results observed in the first fetal oPAD cultured cells. We also tested our optimized adipogenic differentiation system in three different ovine adult primary cultured preadipocytes. Adult subcutaneous preadipocytes were used and a similar differentiation rate to that seen in fetal oPADs was observed (Fig. 2a and b3). Because PPARγ agonists can induce lipid droplet accumulation in non-adipogenic cells, we used ovine fetal skin fibroblasts to test if rosiglitazone could induce lipid droplet accumulation. Fetal fibroblasts originated from the same animals as the oPADs. When subjected to the final protocol (DM2a for 8 days), fetal fibroblasts were not able to accumulate lipid droplets (Fig. 3c).

**Fig. 5:**
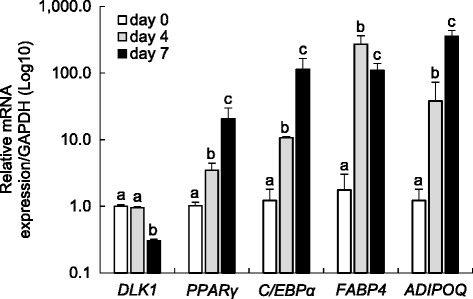
Dynamic gene expression in fetal ovine preadipocytes (oPADs) during differentiation. mRNA expression (mean ± SEM) through adipogenic differentiation in fetal oPADs from day 0 to 7 of differentiation when using the optimized differentiation protocol (Fig. 2b2). Different letters represent significant differences (P < 0.05) within gene and between culture days by ANOVA. Three fetal oPAD primary cells (passage 3) from three different fetuses were used. Gene expression was validated using three housekeeping genes (*GAPDH*, *RPL27*, and *β-actin*), but only *GAPDH* results are shown

To further assess the differentiation process in oPADs, we evaluated the expression of genes expressed in preadipocytes (*DLK1*) and in the commitment (*C/EBPα* and *PPARγ*) and terminal phase (*FABP4* and *ADIPOQ*) of adipogenesis by qRT-PCR. Differentiation was confirmed by a downregulation of *DLK1* and exponential upregulation of *C/EBPα, PPARγ, FABP4*, and *ADIPOQ* (Fig. 5) upon differentiation progression.

In conclusion, our protocol enables successful adipogenic differentiation of fetal and adult ovine preadipocytes. It also demonstrates that compared to the 3T3-L1 cell line, fetal ovine preadipocytes require a longer exposure to the differentiation cocktail, and suggests the need for IMBX, dexamethasone, and/or the PPARγ agonist rosiglitazone through the terminal differentiation phase. They also require higher insulin concentration during differentiation to enhance lipid accumulation and similar to human primary preadipocytes [21, 22], PPARγ agonist supplementation is also required for ovine adipogenic differentiation. This work highlights species-specific differences requirements for adipogenic differentiation [7, 14, 31, 34] and the need to develop standardized methods to investigate comparative adipocyte biology.

## Abbreviations

*ADIPOQ*: adiponectin
ATCC: American Type Culture Collection
BS: Basal medium
*C/EBP*: CCAAT/enhancer-binding protein
Dex: Dexamethasone
*DLK1*: Delta like non-canonical Notch ligand 1
DM: Differentiation medium
DMEM: Dulbecco’s modified Eagle’s medium
DPBS: Dulbecco’s phosphate-buffered saline
*FABP4*: Fatty acid binding protein 4
*GAPDH*: Glyceraldehyde 3-phosphate dehydrogenase
*GPDH*: Glycerol-3-phosphate dehydrogenase
IBMX: 3-isobutyl-1-methylxanthine
Ins: Insulin
oPAD: Ovine fetal preadipocytes
ORO: Oil Red O
*PPARγ*: Peroxisome proliferator-activated receptor γ
qRT-PCR: Quantitative real time PCR
Rosi: Rosiglitazone
*ZFP423*: Zinc finger protein 423
